# A biomaterials approach to influence stem cell fate in injectable cell-based therapies

**DOI:** 10.1186/s13287-018-0789-1

**Published:** 2018-02-21

**Authors:** Mahetab H. Amer, Felicity R. A. J. Rose, Kevin M. Shakesheff, Lisa J. White

**Affiliations:** Centre for Biomolecular Sciences, School of Pharmacy, University of Nottingham, Nottingham, UK

**Keywords:** Biomaterials, Cell therapy, Cell fate, Differentiation, Injectable, Mesenchymal stem cells

## Abstract

**Background:**

Numerous stem cell therapies use injection-based administration to deliver high-density cell preparations. However, cell retention rates as low as 1% have been observed within days of transplantation. This study investigated the effects of varying administration and formulation parameters of injection-based administration on cell dose recovery and differentiation fate choice of human mesenchymal stem cells.

**Methods:**

The impact of ejection rate via clinically relevant Hamilton micro-syringes and biomaterial-assisted delivery was investigated. Cell viability, the percentage of cell dose delivered as viable cells, proliferation capacity as well as differentiation behaviour in bipotential media were assessed. Characterisation of the biomaterial-based cell carriers was also carried out.

**Results:**

A significant improvement of *in-vitro* dose recovery in cells co-ejected with natural biomaterials was observed, with ejections within 2% (*w*/*v*) gelatin resulting in 87.5 ± 14% of the cell dose being delivered as viable cells, compared to 32.2 ± 19% of the dose ejected in the commonly used saline vehicle at 10 μl/min. Improvement in cell recovery was not associated with the rheological properties of biomaterials utilised, as suggested by previous studies. The extent of osteogenic differentiation was shown to be substantially altered by choice of ejection rate and cell carrier, despite limited contact time with cells during ejection. Collagen type I and bone-derived extracellular matrix cell carriers yielded significant increases in mineralised matrix deposited at day 21 relative to PBS.

**Conclusions:**

An enhanced understanding of how administration protocols and biomaterials influence cell recovery, differentiation capacity and choice of fate will facilitate the development of improved administration and formulation approaches to achieve higher efficacy in stem cell transplantation.

**Electronic supplementary material:**

The online version of this article (10.1186/s13287-018-0789-1) contains supplementary material, which is available to authorized users.

## Background

To date, most clinical trials employing cell-based therapeutics have used injectable delivery of cellular suspensions in saline vehicles [1–3]. However, cell loss has been observed within the first minutes post injection [4], with less than 10% of injected cells retained at the site of interest [2, 5]. Consequently, increasing the percentage of viable cells delivered and retained post injection is critical to the future success of cell transplantation procedures.

Mesenchymal stem cells (MSCs) have been the focus of numerous pre-clinical and clinical cell therapy studies [6]. Stem cell fate is regulated by biochemical and biophysical cues from the microenvironment [7, 8]. As such, an intricate range of environmental effectors, experienced both during the delivery procedure and post transplantation, can impact cell function [9]. For example, cells experience various types of mechanical forces, including shear forces, during flow through a needle [10]. Although shear stress has been reported to directly influence the fate of undifferentiated stem cells [11, 12], the impact of mechanical forces experienced during clinically relevant injection procedures upon the differentiation potential of hMSCs has yet to be investigated.

Numerous studies have highlighted that the material surrounding a stem cell is vital in determining its fate [13–15]. Biomaterials can be used as cell delivery vehicles to provide physical support and protection for cells and stimulate host cell recruitment and differentiation [16]. Naturally derived biomaterials may exhibit inherent bioactivity that can facilitate tissue integration. Examples of naturally derived biomaterials include collagen, which has been shown to support cell growth in 3D cell culture studies and *in vivo* [17–21], and tissue-derived extracellular matrices (ECMs), harvested by decellularisation of mammalian tissues [22]. ECM materials retain the inherent bioactivity of the native matrix and modulate cell behaviour and promote constructive remodelling *in vivo* [23]. Other natural biomaterials, such as protein-based polymers, have found utility as cell carriers because these biomaterials may mimic characteristics of the natural ECM and influence the growth and fate of transplanted cells [24]. An example of naturally derived biomaterials is carboxymethyl cellulose (CMC), a biodegradable polysaccharide-based polymer with excellent biocompatibility [25, 26].

With the rising number of clinical trials exploring MSC-based cell therapies, an understanding of the factors that influence the functionality of cells post injection is critical. Despite the advantages of biomaterials as cell transplantation vehicles, saline-based cell carriers still continue to be the carrier of choice for many cell therapy clinical trials [1–3]. Since physical, chemical and biological factors have an impact on differentiation behaviour of cells [27], cues caused by variations in cell administration protocols can contribute to differentiation commitment decisions of MSCs. Our previous work provided evidence that ejection of cell suspensions at a low flow rate negatively impacted cell dose recovery, viability and function [28, 29]. An enhanced understanding of how injectable biomaterials improve cell dose recovery and influence stem cell differentiation will facilitate the development of improved administration and formulation approaches to achieve higher efficacy and reduce variability in stem cell transplantation. The present study aimed to examine the influence of varying cell administration and formulation parameters on fate choice of hMSCs by assessing the impact of ejection upon the differentiation capacity of primary human MSCs using clinically relevant needles and by determining the potential value of user-friendly injectable biomaterials to improve delivery efficiency and to direct cell fate.

## Methods

### Overall experimental design

The general experimental design for this study is depicted schematically in Additional file 1: Figure S1. The first part of this study aimed to determine whether the initial cell seeding density influenced differentiation capacity. This was key to understanding whether any impact observed on differentiation capacity would be related to the number of cells being ejected at the slow flow rates employed [28] or to the effect of cell administration variables under investigation. The second part of the study assessed the impact of varying ejection rate on the differentiation capacity of ejected cells. Cell dose recovery and differentiation capacity of hMSCs ejected within various injectable biomaterial-based carriers were examined at low ejection rates. Differentiation to osteoblastic and adipogenic lineages was examined in bipotential differentiation ‘mixed’ media, with a formulation designed to induce both.

### Human mesenchymal stem cell culture

Primary human bone marrow mesenchymal stem cells (hMSCs) were obtained from Lonza and cultured in mesenchymal stem cell growth medium (MSCGM) (#PT-3001; Lonza, Cologne, Germany) with 5% CO_2_ in air at 37 °C. Lot numbers of hMSC batches obtained were #0000351482, #0000411107 and #0000422610, cultured as individual patient stocks. Cells used in this study were between the third and fifth passages. These cells were tested for the ability to differentiate into osteogenic, adipogenic and chondrogenic lineages, and for expression of surface markers recommended by the International Society for Cellular Therapy (ISCT) [30]. All routine passaging and differentiation procedures were performed according to Lonza’s Poietics™ hMSC protocols.

### Effects of cell seeding density on differentiation potential of hMSCs

Cell seeding densities tested ranged from 1000 to 70,000 hMSCs per well in 12-well plates (Nunc, Thermo Fisher Scientific, UK), equivalent to 0.3 × 10^3^–18.4 × 10^3^ cells/cm^2^. Twenty-four hours after seeding, the medium was replaced with bipotential differentiation medium, formulated by combining adipogenic (#PT-3004; Lonza) and osteogenic (#PT-3002; Lonza) media in a 1:1 ratio. The culture was incubated for 21 days. The differentiation medium was changed every 3–4 days for the duration of the differentiation period. Nuclear-based cell counts were carried out using propidium iodide (PI) staining of the fixed cells in osteogenesis experiments and using Hoechst 33258 in adipogenesis studies.

### Preparation of hMSCs and ejection protocol

After trypsinisation, cells were centrifuged and then reconstituted to a density of 1.4 × 10^6^ cells/ml in phosphate buffered saline (PBS) for cell ejection studies, and then were mixed 1:1 with either PBS or the biomaterial-based carrier under investigation (via gentle mixing) to a final density of 7 × 10^5^ cells/ml. Density of cell suspensions used in this study was selected conservatively based on published clinical studies [31–33] as well as practical considerations. Aliquots (100 μl) of this cell suspension were used for ejection experiments. Cells were directly pipetted into well plates (not ejected) to provide controls. For ejection, 100-μl Hamilton GASTIGHT^®^ syringes (model 1710RN) attached to 30G 20-mm removable (RN) stainless steel needles were employed (Hamilton Bonaduz, Switzerland). All ejection studies were carried out at room temperature. Cell/carrier mixtures were set aside for 20 min before ejection, to represent the delay time between loading and injection in cell transplantation procedures [34]. Ejection rates employed in clinical trials have been variable, ranging from 5 μl/min [35] to 1000 μl/min for stroke [36, 37]. Therefore, ejection rates employed in this study (10–300 μl/min) were selected to represent a range of clinically relevant ejection rates used previously in clinical trials. Cell suspensions were drawn up with a Harvard^®^ Infuse/Withdraw syringe pump (PHD 2000; Harvard Apparatus^®^, MA, USA) at 300 μl/min through the needle before being ejected at the chosen flow rate into Eppendorf tubes. Ejected samples were then transferred into the appropriate well plates.

### Qualitative and quantitative assessment of differentiation capacity of ejected hMSCs

Differentiation to osteoblastic and adipogenic lineages was examined in bipotential ‘mixed’ media (see ‘Effects of cell seeding density’). Bipotential medium was added to the cells in 12-well plates at 24 h post seeding, and cells were alternated between adipogenic induction/osteogenic induction media (1:1) and adipogenic maintenance/osteogenic media (1:1) every 3–4 days, according to the manufacturer’s instructions, for 21 days. Uninduced controls were maintained in basal medium MSCGM (#PT-3001; Lonza).

#### Adipogenic differentiation

After 21 days, cultures were rinsed with 70% (v/v) isopropanol for 5 min and differentiation was assessed qualitatively by specific staining of lipid droplets with 0.5% Oil Red O solution (Sigma-Aldrich, Poole, UK). Intracellular lipid accumulation was quantified using the AdipoRed™ Adipogenesis Assay (Lonza) following the manufacturer’s protocol. Briefly, cells were pre-washed with PBS and incubated with AdipoRed™ Reagent for 10 min. Fluorescence was measured using a plate reader (λ_exc_/λ_em_ 485/572 nm).

#### Osteogenic differentiation

After 21 days of incubation, cells were fixed in 10% (v/v) formalin. The presence of extracellular calcium deposits was qualitatively verified using Alizarin Red staining solution (Merck Millipore, UK) and von Kossa silver staining kit (Merck Millipore). For Alizarin Red staining, cells were treated with Alizarin Red S for 5 min at room temperature. After washing three times in deionised water, cells were observed microscopically. For von Kossa staining, cells were incubated with silver nitrate solution under exposure to UV light for 20 min. Wells were then washed with deionised water three times, and treated with sodium thiosulfate solution for 5 min. Afterwards, wells were washed three times with deionised water. Mineralised nodules were visualised as black spots.

##### OsteoImage™ staining for quantitation of hydroxyapatite deposition

*In-vitro* mineralisation was assessed by staining the hydroxyapatite portion of mineralised bone nodules using the OsteoImage™ Mineralisation Assay kit (Lonza) according to the manufacturer’s instructions. Briefly, cells were fixed in 10% (v/v) formalin, rinsed twice using the wash buffer, stained with the staining solution provided (1:100 dilution in wash buffer) and incubated for 30 min. Following incubation, cells were rinsed three times with wash buffer. This assay allows the assessment of *in-vitro* mineralisation both visually by fluorescent microscopy and quantitatively using a plate reader (λ_exc_/λ_em_ 492/520 nm).

##### Quantitative alkaline phosphatase staining

For quantitative determination of alkaline phosphatase (ALP) activity, the Fluorometric Alkaline Phosphatase Assay Kit (#ab83371; Abcam, UK) was used, as per the manufacturer’s protocol. Cultured cells were lysed using three freeze–thaw cell lysis steps. Samples (media or cell lysate) were incubated with the non-fluorescent 4-methylumbelliferyl phosphate disodium salt (MUP) as a substrate. The resultant fluorescence was measured using a plate reader. ALP activity was normalised to total DNA content, measured using the Quant-iT PicoGreen dsDNA Assay Kit (#P11496; Invitrogen, UK).

##### Osteocalcin immunostaining

hMSCs were rinsed with warm PBS and fixed with 3.7% (*w*/*v*) paraformaldehyde in PBS for 20 min, followed by a wash with warm PBS for 5 min. Cells were permeabilised using 0.1% (w/v) Triton-X 100 in PBS (Sigma-Aldrich) for 30 min. Non-specific binding sites were blocked by incubation in 10% (*v*/v) normal donkey serum (D9663; Sigma-Aldrich) and 1% bovine serum albumin (BSA) in PBS for 1 h. Cells were then incubated with Mouse Anti-Human Osteocalcin Monoclonal Antibody (MAB1419; R&D Systems) diluted in 1% (w*/*v) BSA in PBS at 10 μg/ml for 3 h at room temperature. This was followed by two washes with PBS supplemented with 0.1% (w/v) BSA (5 min each). The secondary antibody, a Donkey Anti-Mouse IgG-FITC antibody (NL007; R&D Systems), diluted in 1% (w/v) BSA in PBS (1:200) was added for 1 h in the dark, followed by two washes with PBS (5 min each). Samples were counterstained with DAPI NucBlue^®^ Fixed Cell ReadyProbes (ThermoFischer Scientific) for 5 min, and then visualised using a Leica DM-IRB inverted microscope (Leica Microsystems Ltd., UK).

### Preparation of biomaterial-based carriers for ejection of hMSCs

#### Carboxymethyl cellulose

CMC carriers were prepared using high-viscosity CMC (#12M31P; Ashland Speciality Ingredients, Poole, UK) in tissue culture water at concentrations of 0.5% and 0.25% (*w*/*v*). CMC carriers were sterilised by tyndallisation, carried out by heating the prepared solutions three times at 70 °C for 20 min each at 24-h intervals.

#### Type I collagen

Commercially available high-concentration rat-tail collagen type I (#354249, 10 mg/ml; BD Biosciences, Oxford, UK) was prepared at a concentration of 1.75 mg/ml following the manufacturer’s instructions. Briefly, collagen was freshly prepared for experiments by combining rat-tail collagen type I with 10× PBS, ice-cold 1 N sodium hydroxide and sterile ice-cold deionised water to achieve a final collagen concentration of 1.75 mg/ml. The solution was mixed and kept at 4 °C.

#### Bone extracellular matrix

Decellularised and demineralised bone extracellular matrix (bECM) was obtained as described previously [38]. Briefly, liquid nitrogen was used to freeze bovine cancellous bone to facilitate fragmentation. Cancellous fragments were demineralised using 0.5 M HCl at room temperature for 24 h. Following demineralisation, a solution of chloroform/methanol was used to remove lipids and then demineralised powder was subjected to 24 h of decellularisation in 0.05% Trypsin/0.02% EDTA at 37 °C. Powdered bone was combined with 1 mg/ml pepsin in 0.01 M HCl for a final concentration of 10 mg/ml and this suspension was stirred for 96 h. Resultant bECM digests were aliquoted and stored at − 20 °C until required. Neutralisation of the required digest volume was carried out by addition of one-tenth of the digest volume of NaOH (0.1 N) and one-ninth of the digest volume of PBS (10×), and then diluting to the desired final bECM concentration with 1× PBS on ice. A concentration of 1.75 mg/ml bECM was freshly prepared for ejection studies.

#### Gelatin

Commercially available 2% (w/v) gelatin solution in water, derived from bovine skin (type B, #G1393; Sigma-Aldrich), was used for biomaterial-based cell ejection studies.

### Assessing cell recovery and proliferation using PrestoBlue™

Since cell dose recovery results determined using PrestoBlue™ were shown previously to be comparable to the DNA-based Cyquant assay for determination of cell numbers ejected through Hamilton syringes [28]. PrestoBlue™ (Invitrogen Life Sciences, Paisley, UK) was used to determine 24-h viability following ejection of cell suspensions (7 × 10^5^ cells/ml), as well as proliferation over several days. The PrestoBlue™:culture medium (1:9) mixture was added to each well, and incubated in the dark at 37 °C for 1 h. Triplicate 100-μl aliquots were measured for fluorescence on a Tecan Infinite M200 microplate reader (Tecan, Reading, UK) at λ_exc_/λ_em_ 560/590 nm.

### Multiplexing quantitative differentiation assays with nuclear staining

Multiplexing of cell-specific differentiation assays and nuclear staining allowed for normalisation to cell number. After differentiated cells were stained with the OsteoImage™ Staining Reagent, 100 μg/ml RNase A solution (AppliChem, Darmstadt, Germany) in Tris–EDTA buffer solution (Sigma-Aldrich) was added for 10 min at room temperature. Nuclear staining was carried out using 2 μg/ml PI (diluted in H_2_O from 1 mg/ml; ThermoFisher Scientific, UK) for 5 min. For quantitation, the mean fluorescence intensity of each well was determined using multiple readings of each well at λ_exc_/λ_em_ 490/530 nm for OsteoImage™ (> 64 readings/well) and 535/617 nm for PI (100 readings/well). For quantification of adipogenesis, differentiated cells were stained with AdipoRed™ Assay Reagent (Lonza). Nuclear staining in adipogenic analysis was carried out using 2 μg/ml Hoechst 33,258 (diluted in H_2_O from 1 mg/ml; Sigma-Aldrich) for 15 min. The mean fluorescence intensity of each well was determined using 100 readings per well.

### Rheological analysis of biomaterial-based carriers

Rheological assessment was carried out with a Physica MCR301 rheometer (Anton Paar, UK) using rotational and oscillatory measurements. A 50-mm diameter cone plate (CP 50–1) was used, except for thixotropy recovery studies which were performed using a PP 25 parallel plate, with a 0.2-mm measuring gap. All measurements were carried out at a controlled temperature of 25 °C. Samples were allowed to equilibrate for at least 2 min prior to analysis. A minimum of three independent measurements were obtained for each sample, and the average value was reported. Viscosity was determined using a constant shear rate of 1 s^− 1^. The average value of all readings at 6-s intervals over a span of 120 s was taken as the viscosity measurement. Steady shear rheology was performed with a shear rate varying from 0.01 to 1000 s^− 1^. Viscometric thixotropy testing was carried out by applying a high-magnitude strain (10,000 s^− 1^) to break the biomaterial’s structure, followed by a low-level strain (1 s^− 1^) to observe the rate and extent of recovery of carrier bulk properties. Samples were also subjected to oscillatory strain sweeps from 0.1 to 1000% performed at 6 rad/s to assess the failure strain for these materials.

### Contact angle measurement

Contact angles of the carriers with glass were measured using the sessile drop method. Soda-lime glass slides were used for measuring contact angles, which have similar wettability and surface tension in air to the borosilicate glass surfaces of syringes [39]. Contact angles were measured with a CAM 200 instrument (KSV Instruments, Finland) after 10-s spreading time. A drop of the material to be tested was formed on the end of a precision syringe and placed onto the glass slide. Ten images of the drop were taken at 1-s intervals. All measurements were made at 25 °C. The contact angle was calculated for each image using a Young–Laplace curve fit using the CAM 200 image analysis software, and resulting right and left contact angles were averaged. A minimum of five repeat measurements were made for each material using separate glass slides.

### Statistical analysis

Statistical analyses were performed using GraphPad Prism 6 software. Data sets were tested for normality and suitable tests of comparisons were subsequently chosen. All values were reported as mean value ± SD, unless stated otherwise. Data were analysed by one-way or two-way analysis of variance (ANOVA) with Dunnett’s *post-hoc* test, unless otherwise stated. *p* ≤ 0.05 was considered significant.

## Results

The first part of this study determined whether the initial cell seeding density influenced differentiation capacity. Previously, we showed that the ejection rate affected the cell dose delivered and that cell recovery was negatively impacted by low ejection flow rates [28, 29]. Thus, it was critical to quantitatively assess the impact of the cell dose delivered upon the differentiation capacity. The second part of the study assessed the impact of varying ejection rate on the differentiation behaviour of ejected cells. Lastly, biomaterial-based formulations were selected as candidate biomimetic carriers to maximise hMSC recovery at low ejection rates and investigate the differentiation behaviour of ejected cells.

### Impact of initial cell seeding density on differentiation potential of hMSCs

Hydroxyapatite deposition was used as a marker of osteogenic differentiation of hMSCs. Additional file 2: Figure S2A, B shows no significant difference in osteogenic differentiation between the various cell numbers seeded initially, as shown by amounts of hydroxyapatite deposited and normalised mineral deposition to cell number (*p* < 0.05). All cell densities exhibited similar final cell numbers at day 21, with lower initial cell seeding densities exhibiting a significantly higher fold change in cell number relative to day 0 (*p* < 0.05) when expressed as fold change in cell number relative to the number of cells initially seeded (Additional file 2: Figure S2C). There was no significant difference in the osteogenic differentiation of different cell seeding densities, as shown by fluorescence microscopy images of hydroxyapatite bone nodules (Additional file 2: Figure S2D).

In contrast to this, there was a clear correlation between initial seeding density and adipogenesis, as shown by AdipoRed™ staining and statistically significant differences in normalised fluorescence data relative to the full seeding density of 7 × 10^5^ cells per well (*p* < 0.05; Additional file 3: Figure S3A, B). The dependence of adipogenesis on initial cell seeding density is also shown in Additional file 3: Figure S3C, where fluorescence microscopy images demonstrate an increasing intensity of fluorescent staining of intracellular lipids with increasing initial cell seeding numbers.

The study therefore focused on osteogenesis for subsequent studies as a model of differentiation to investigate the impact of ejection rate on differentiation of ejected hMSCs, since mineralisation at day 21 was shown to be independent of the initial cell seeding density.

### Ejection rate influenced osteogenic differentiation of ejected hMSCs

**Fig. 1:**
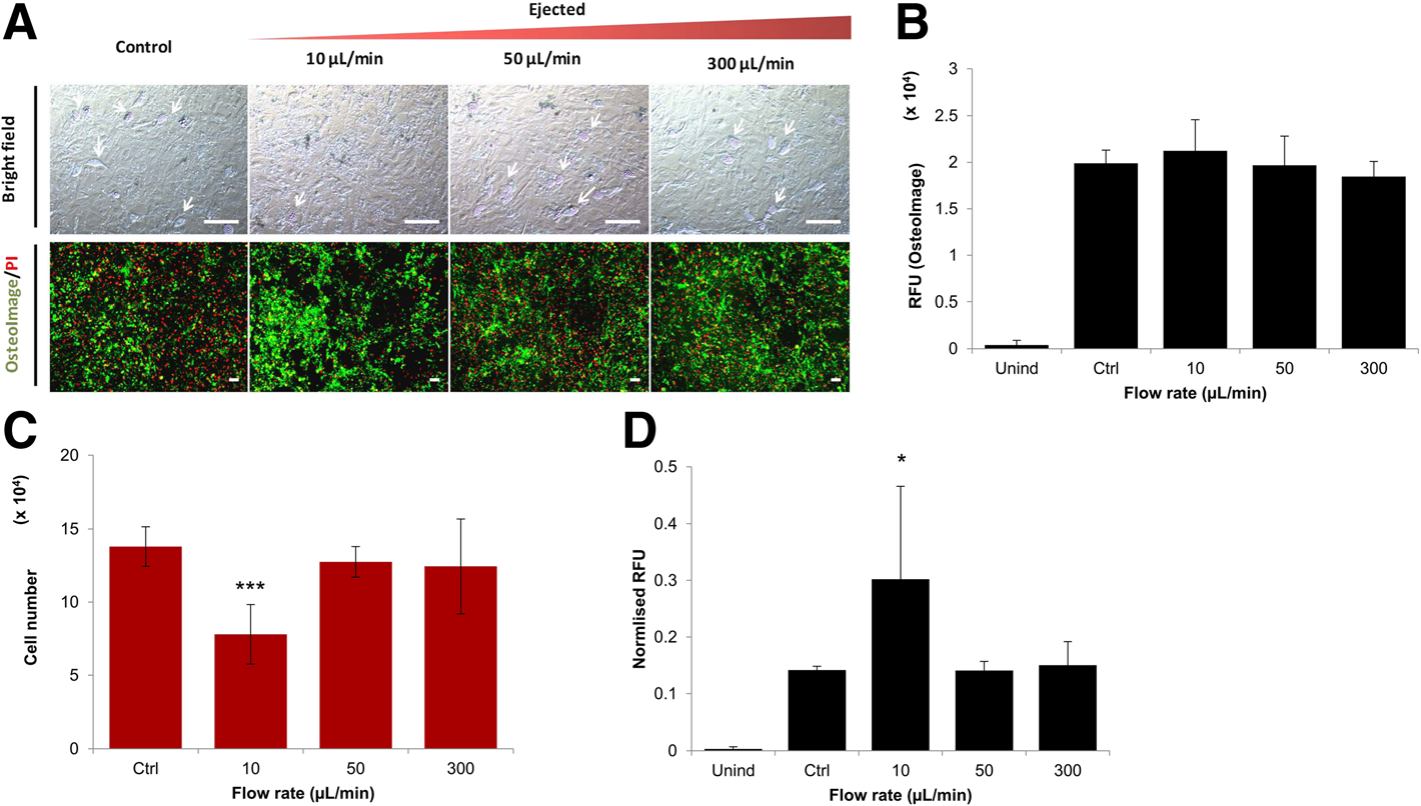
Effect of ejection rate on osteogenic differentiation capacity of hMSCs ejected via 30G needles, cultured in bipotential osteogenic/adipogenic media for 21 days. **a** Representative bright-field and fluorescence microscopic images showing ejected hMSCs after culturing in bipotential media. Cell nuclei stained red (PI), mineralised areas stained green (OsteoImage™). Intracellular lipid accumulation indicated by arrows (scale bar = 100 μm). **b** OsteoImage™ fluorescence values for ejected hMSCs at day 21. Each bar represents mean fluorescence values ± SD (*n*  =  9 in three independent experiments and two donors). **c** Number of cells at day 21 quantitated using PI staining of hMSCs from two donors (mean ± SD, *n*  =  6). **d** OsteoImage™ fluorescence readings normalised to cell count, based on PI staining (mean ± SD, *n*  =  6). Statistically significant difference from directly plated control using ANOVA and Dunnet’s *post-hoc* test: *p ≤ 0.05, ***p ≤ 0.001. PI propidium iodide, RFU relative fluorescence unit, Unind uninduced, Ctrl control

Directly plated control and ejected samples showed mixed populations of both adipocytes and osteoblasts. Samples ejected at 10 μl/min exhibited visibly less cells exhibiting the adipocyte morphology compared to other samples. In addition, brighter OsteoImage™ staining of hydroxyapatite (HA) was evident in the samples ejected at 10 μl/min relative to directly plated controls and other ejected samples (Fig. 1a). All ejected samples gave similar absolute fluorescence values when stained with OsteoImage™ (Fig. 1b); samples ejected at 10 μl/min exhibited lower cell numbers at day 21 relative to the control (Fig. 1c). However, samples ejected at 10 μl/min exhibited statistically significant higher normalised fluorescence values (Fig. 1d), compared to the directly plated control at 21 days (*p* < 0.05).

### Biomaterial-based delivery systems enhanced cell dose recovery and viability

**Fig. 2:**
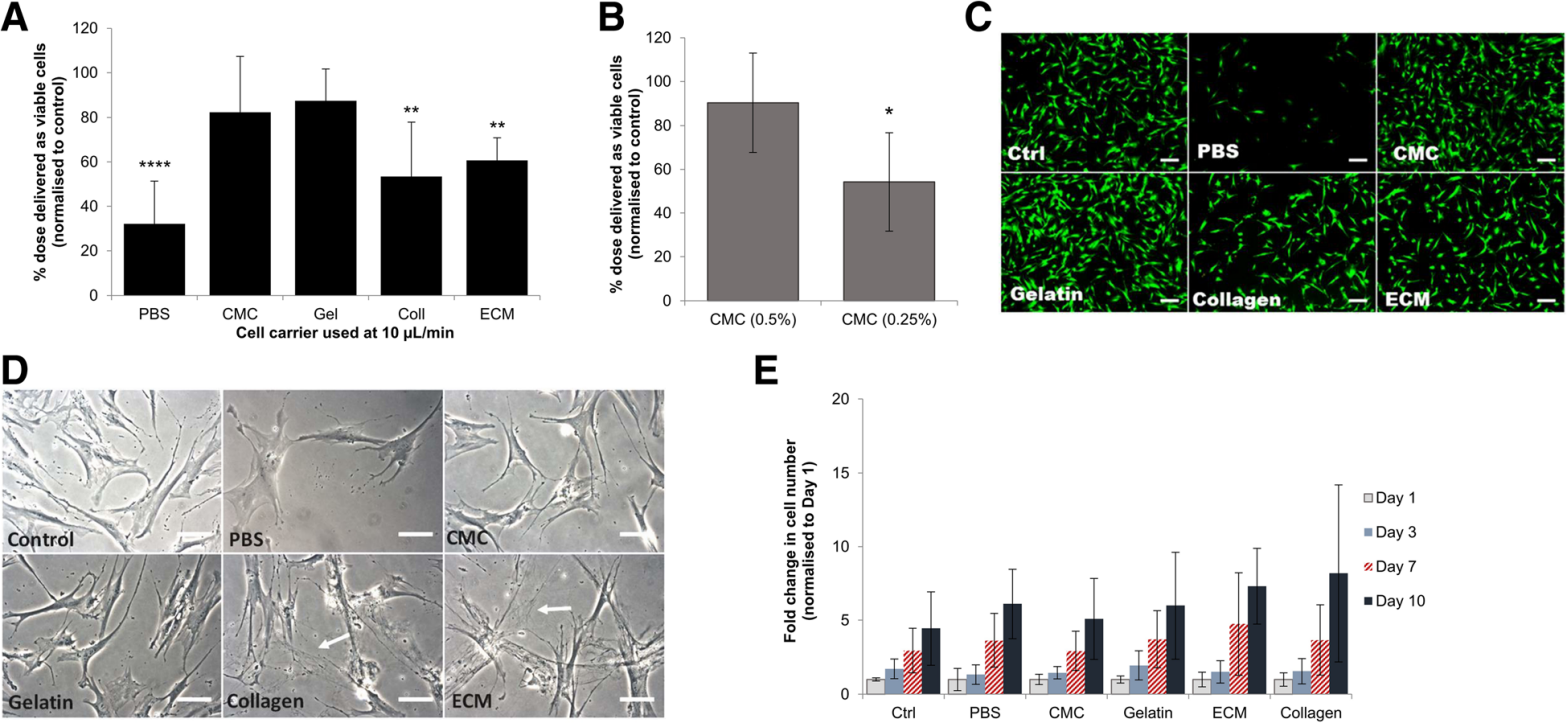
Cell recovery, viability and proliferation after ejecting hMSCs suspended in various carriers via 30G needles at 10 μl/min. **a** Proportion of hMSCs delivered, measured using PrestoBlue™, within phosphate buffered saline (PBS), carboxymethyl cellulose (CMC), gelatin (Gel), type I collagen (Coll) and bone extracellular matrix (bECM). Data represent averages from three donors in five independent experiments (*n* = 5, mean ± SD). Data normalised against control value of directly plated cells. **p ≤ 0.01, ****p ≤ 0.0001, one-way ANOVA with Dunnett’s *post-hoc* test. **b** Percentage of cell dose delivered as viable cells when ejected at 10 μl/min via 30G needles suspended within two concentrations of CMC (0.5% and 0.25%). Data represent averages from two donors (*n* = 3, mean ± SD). Data normalised against control value of directly plated cells. Statistically significant differences between numbers of ejected cells compared with control: *p = 0.05, one-way ANOVA with Kruskal–Wallis analysis. **c** Representative Live/Dead-stained fluorescence images of hMSCs 24 h following ejection at 10 μl/min, using various biomaterials as cell carriers (scale bar = 100 μm). **d** Representative bright-field images showing morphology of ejected hMSCs cultured for 24 h post ejection. Bundles of fibrils surrounding the ejected cells depicted by arrows (scale bar = 50 μm). **e** Proliferation of hMSCs given as fold change in number from day 1 of each sample, measured using PrestoBlue™ (mean ± SD, *n* = 4 measured in two donors). Ctrl control

As retention in the delivery device and shear stress may result in a lower number of viable cells delivered, it was hypothesised that co-injecting cells with biomaterial-based carriers may improve recovery. Collagen type I, gelatin, bone decellularised ECM (bECM) and high-viscosity CMC were selected to test this hypothesis. Incorporating cells in a protective viscous medium improved cell delivery (Fig. 2a). Cells suspended within 2% gelatin type B exhibited the best recovery, with a significantly improved percentage of viable cells of 87.5 ± 14.1% compared to 32.1 ± 19.1% of the dose delivered when ejected with PBS*.* Relative to gelatin and CMC, delivery with collagen and bECM provided significantly lower percentages of viable cells, with 53.4 ± 24.4% and 60.7 ± 10.1% respectively delivered. To investigate the effect of using a lower concentration of the same material, two concentrations of CMC (0.5% and 0.25% (*w*/*v*)) were compared (Fig. 2b). The lower concentration (0.25% (w/v)) of CMC resulted in a significantly lower percentage of the dose being delivered as viable cells (*p* = 0.05).

Live/Dead staining revealed a high proportion of viable cells among all cell carriers investigated (Fig. 2c). However, a visibly lower number of cells appeared in ejected samples suspended in PBS compared with other carriers. Bright-field images of the ejected samples 24 h post ejection (Fig. 2d) displayed an obvious presence of fibril-like structures in samples ejected within collagen and bECM; cells ejected within other carriers did not display fibrillogenesis. These fibrils were not stained by calcein (Fig. 2c). The proliferative ability of ejected hMSCs was not significantly affected by choice of cell carrier, with similar fold changes in cell numbers observed at day 10 relative to the control (Fig. 2e).

### Characterisation of biomaterial-based carriers

**Fig. 3:**
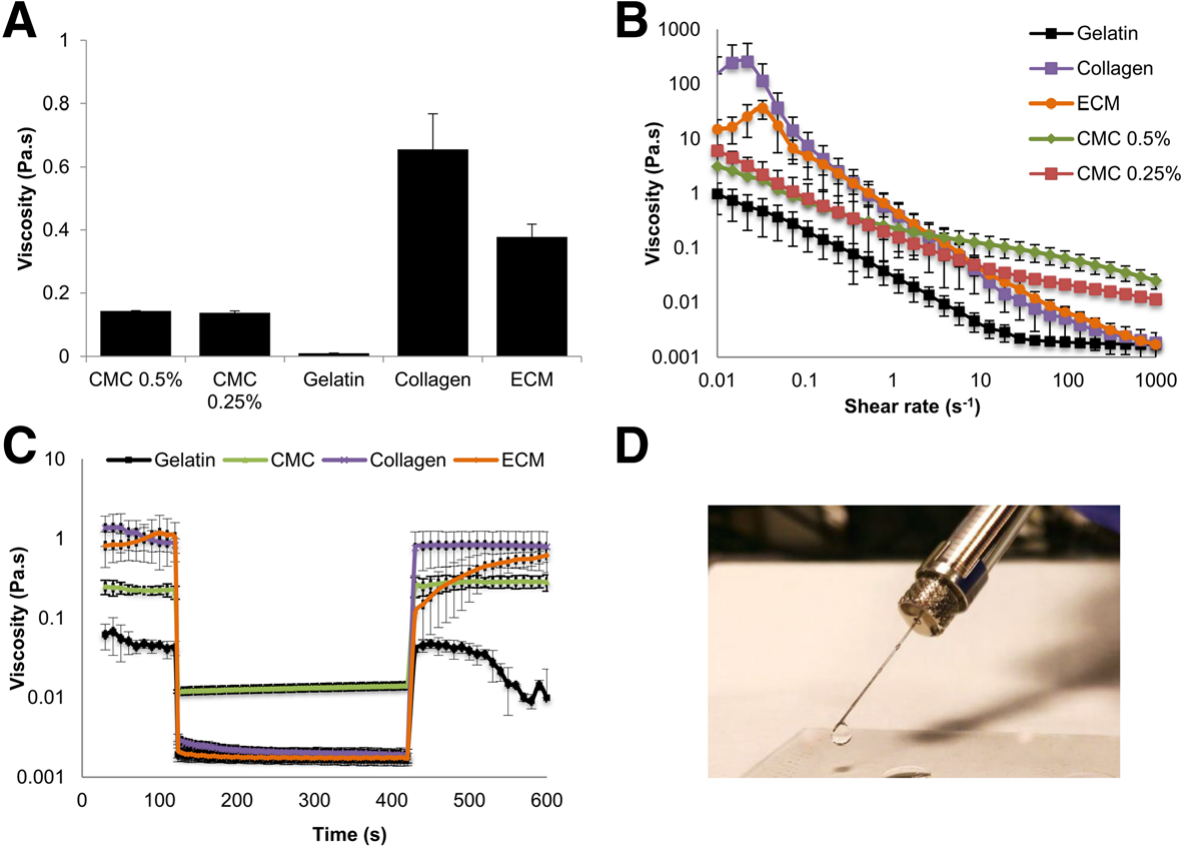
Rheological characterisation of biomaterial-based cell carriers employed in this study. **a** Viscosities of carriers determined at 25 °C with a constant shear rate of 1 s^− 1^ (mean ± SD, *n* = 3). **b** Shear thinning properties of different carriers employed shown by steady shear rheological measurements. **c** Viscometric three-step thixotropy test to display structure recovery of the hydrogel immediately after disruption due to a high-magnitude shear-rate strain (10,000 s^− 1^), followed by a low-magnitude strain (1 s^− 1^) to monitor recovery of bulk properties. **d** CMC 0.5% ejected through a 30G needle, representative of injectability of biomaterials investigated. CMC carboxymethyl cellulose, ECM extracellular matrix

The rheological properties and surface tension of the carriers were characterised to elucidate the effects of material properties upon cell behaviour. Gelatin displayed the lowest viscosity (0.01 ± 0.0007 Pa.s) and collagen the highest (0.66 ± 0.11 Pa.s) (Fig. 3a). The steady shear rheological properties of the carriers are presented in Fig. 3b. bECM, gelatin and collagen showed more significant shear-thinning profiles (Δ*η* ~ 10^4^ Pa.s) than both concentrations of CMC (Δ*η* ~ 10^2^ Pa.s). Collagen had a viscosity of around 150 Pa.s at a shear rate of 0.01 s^− 1^, shearing down to 0.001 Pa.s at 1000 s^− 1^. Gelatin exhibited a viscosity of around 1 Pa.s at 0.01 s^− 1^, shearing down to 0.002 Pa.s at a shear rate of 1000 s^− 1^ and not crossing over the profiles of both CMC concentrations.

A viscometric three-step thixotropy test was carried out to investigate recovery of material properties (Fig. 3c). CMC and collagen showed fast and complete recovery within seconds. Gelatin recovered its viscosity within a few seconds to almost 90% of its initial value, but started breaking down 70 s following strain removal. bECM showed a more gradual recovery to almost 65% of its original properties at 180 s following strain removal. Due to their shear-thinning and self-healing properties, all carriers investigated were shown to be injectable through a clinically relevant 30G needle (Fig. 3d).

All cell carriers employed in this study, except CMC, showed higher storage moduli than loss moduli, with curves being parallel and almost linear, which confirmed their hydrogel nature. However, CMC displayed a higher G′′ than G′, and should therefore be classified as a viscous carrier or viscosity modifying excipient. All carriers displayed broad linear viscoelastic regions (Additional file 4: Figure S4). Collagen and bECM exhibited failure of the gel structure at high strains of around 65% and demonstrated strain stiffening behaviour.

**Fig. 4:**
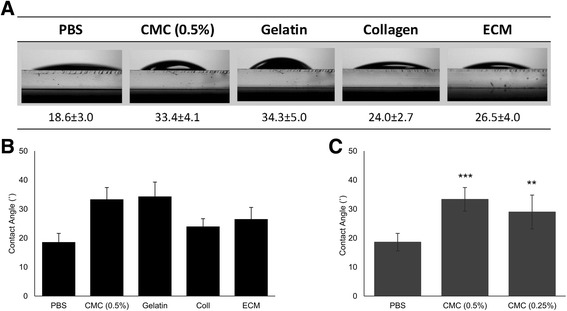
Measurement of contact angles of biomaterial-based cell carriers on a glass surface. **a** Contact angle images of various biomaterials employed on soda-lime glass slides measured at room temperature. **b**, **c** Measurement of contact angles of various carriers on a soda-lime glass surface. Each bar represents mean ± SD (*n* = 5). **p < 0.01, ***p < 0.001, relative to PBS. PBS phosphate buffer saline, CMC carboxymethyl cellulose, Coll collagen, ECM extracellular matrix

All biomaterials exhibited water contact angles ranging from 18° to 35° (Fig. 4a). PBS, collagen and bECM were the most hydrophilic; PBS had the lowest contact angle of 18.6 ± 3.0°, while collagen was less hydrophilic with a contact angle of 24.0 ± 2.7°. Gelatin was the least hydrophilic among the biomaterials investigated, with a contact angle of 34.3 ± 5.0° (Fig. 4b). The more dilute concentration (0.25%) of CMC exhibited a lower contact angle of 29.0 ± 5.8°, compared to 33.4 ± 4.1° for the higher concentration of 0.5% (Fig. 4c).

### Impact of biomaterial-based cell carriers upon differentiation capacity of hMSCs

**Fig. 5:**
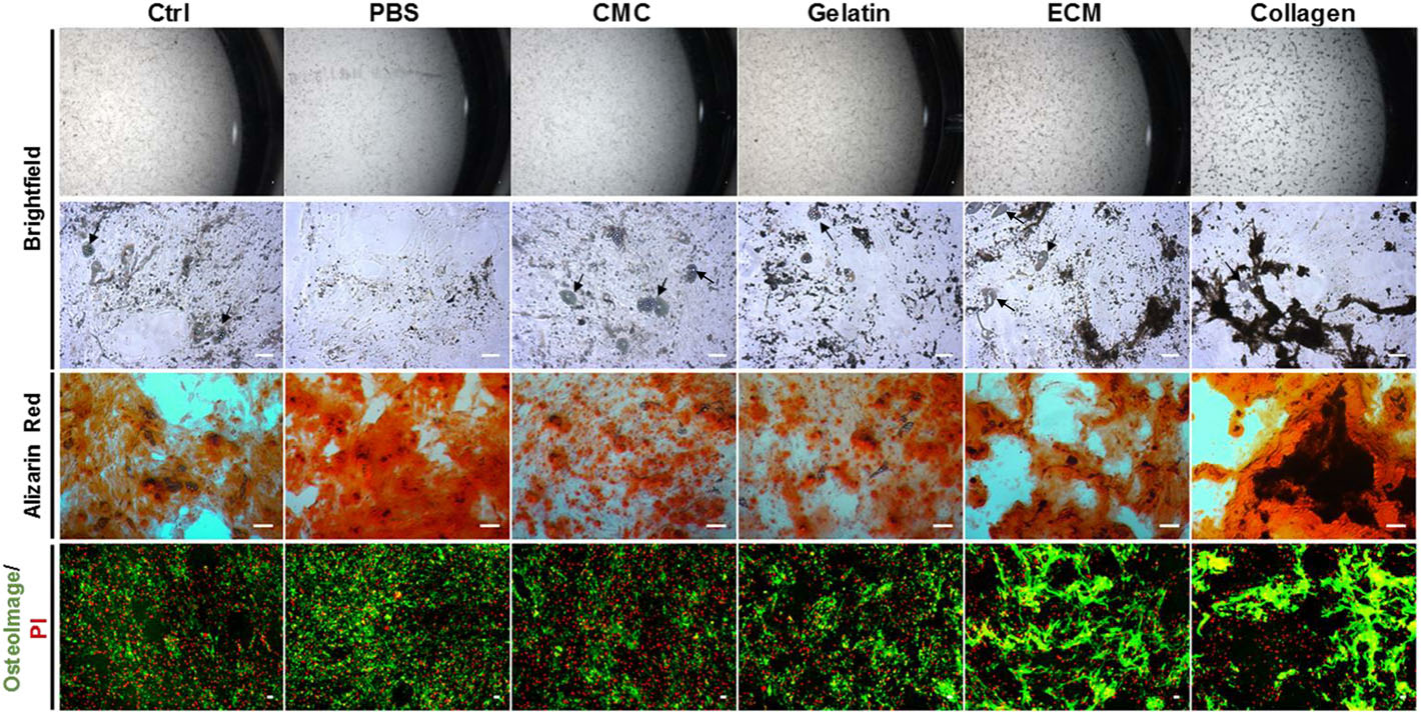
Representative bright-field and fluorescence microscopy images displaying qualitative assessment of osteogenic differentiation capacity of hMSCs post ejection, after culturing in bipotential ‘mixed’ media for 21 days. To assess the degree and distribution of mineralisation of the ECM, the last stage of osteogenesis, samples were observed using dissection and bright-field microscopy (10×), in addition to staining with Alizarin Red S (10×) and OsteoImage™/PI (5×). Cells exhibiting typical adipocyte morphology depicted in bright-field microscopy images using arrows (scale bars = 50 μm). Red represents calcium deposition stained using Alizarin Red, and green depicts hydroxyapatite nodules stained using OsteoImage™. Ctrl control, PBS phosphate buffer saline, CMC carboxymethyl cellulose, ECM extracellular matrix, PI propidium iodide

**Fig. 6:**
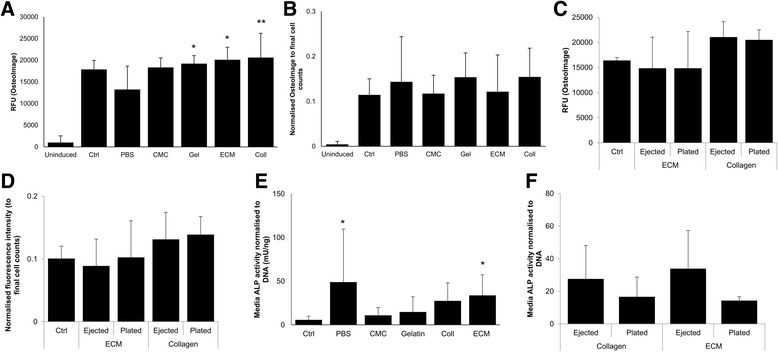
Impact of biomaterial-based carriers on osteogenic differentiation markers of hMSCs ejected via 30G needles, cultured in bipotential media for 21 days. **a** OsteoImage™ fluorescence values, showing hydroxyapatite formation. Each bar represents mean fluorescence value ± SD, *n*  =  6 in three donors. Statistically significant difference relative to PBS using ANOVA and Dunnet’s *post-hoc* test: *p ≤ 0.05, **p ≤ 0.01. **b** OsteoImage™ fluorescence intensity readings normalised to PI-based cell counts (mean ± SD, *n*  =  6). **c** Ejected versus directly plated hMSCs, suspended within collagen and ECM, assessed for osteogenic differentiation capacity. ‘Ejected’ cells were ejected at 10 μl/min, and ‘plated’ cells were 60% of ejected cell number directly plated in 12-well plates. Results are mean fluorescence values ± SD, *n*  =  4 in two donors. (**d**) OsteoImage™ fluorescence intensity readings in (**c**) normalised to PI-based cell counts (mean ± SD, *n*  =  4 in two donors). **e** Media alkaline phosphatase (ALP) activity levels of hMSCs ejected at 10 μl/min, via 30G needles, cultured in bipotential osteogenic/adipogenic media for 2 days after adding differentiation media 24 h post ejection. Values shown are mean ± SD (*n* = 3 in two donors). Statistically significant differences in ALP levels relative to control (Friedman test with Dunn’s *post-hoc test)*: *p < 0.05. **f** Released ALP levels in ejected versus directly plated hMSCs suspended within collagen and ECM. ‘Ejected’ cells were ejected at 10 μl/min, and ‘plated’ cells were 60% of the initially ejected cell number directly plated in 12-well plates. Results are mean fluorescence values ± SD (*n*  =  4 in two donors). RFU relative fluorescence unit, Ctrl control, PBS phosphate buffer saline, CMC carboxymethyl cellulose, ECM extracellular matrix, Coll collagen

All directly plated control and ejected hMSCs underwent osteogenic differentiation after 21 days of culture in bipotential medium (Fig. 5). All samples exhibited a mixed population of cells displaying typical adipocyte morphology as well as mineralised bone nodules. PBS-ejected samples consistently displayed visibly lower numbers of cells exhibiting adipocyte morphology, in addition to some cells displaying a fibroblast-like morphology typical of undifferentiated MSCs. Whilst fluorescence images indicated that all control and ejected samples exhibited mineralisation, a notably higher extent of calcium and hydroxyapatite deposition was observed in samples ejected within collagen and bECM compared to the control and other carriers. Quantitative assessments of hydroxyapatite deposition (Fig. 6a) confirmed that cells co-ejected within collagen, bECM and gelatin led to significantly enhanced mineralised content relative to samples ejected within PBS. However, all samples exhibited no significant difference in normalised fluorescence values (Fig. 6b).

To determine whether ejection forces and biomaterial-based delivery had a synergistic effect upon mineralisation, hydroxyapatite deposition was compared in cells suspended within collagen and bECM, either directly plated in 12-well plates (referred to as ‘plated’) or ejected at 10 μl/min (‘ejected’). Samples were directly plated at 60% of the initially ejected cell number within each carrier material to account for the loss of cells during ejection and to preclude any potential influence of initial seeding density (Fig. 2a). As shown in Fig. 6c, d, there was no significant difference in hydroxyapatite deposition between the directly plated and ejected cells within collagen and bECM at day 21, in terms of both absolute and normalised values.

To determine whether ALP was released into the media as a result of shear stress exposure, culture media were assayed following induction of differentiation. At day 2, media ALP activity was generally higher in ejected hMSCs compared to directly plated samples. Ejection within PBS and bECM increased released ALP activity compared with directly plated hMSCs (Fig. 6e, *p* < 0.05). To determine whether ejection forces and biomaterial-based carriers had a synergistic effect upon released ALP, media ALP levels in plated versus ejected cells, suspended within collagen or bECM, were compared. Figure 6f shows increased media ALP levels in ejected samples relative to plated ones within collagen and bECM.

Although total cellular ALP activity was significantly lower for hMSCs ejected within PBS than for control samples at day 2 (Additional file 5: Figure S5A), normalised levels of ALP production were similar for all samples tested at this time point (Additional file 5: Figure S5B). Expression of normalised cellular ALP generally peaked earlier (day 4) for directly plated samples compared to ejected samples (day 7). To explore whether shear forces contributed to ALP expression pattern, directly plated and ejected hMSCs within bECM and collagen carriers were compared. The same pattern of normalised ALP levels peaking earlier (at day 4) in directly plated samples relative to ejected samples was observed (Additional file 5: Figure S5C), and no significant differences in DNA content (Additional file 5: Figure S5D). After 21 days, immunostaining for osteocalcin (OCN) showed a robust expression of this late osteogenic marker across all samples after 21 days of culture (Additional file 5: Figure S5E).

## Discussion

Since previous work demonstrated that cell ejections at slower flow rates resulted in lower percentages of the cell dose being delivered [28, 29], experiments were carried out first to investigate the dependence of differentiation capacity on initial cell number using bipotential media. In typical monopotential differentiation media, two different variables may act to direct hMSCs down a certain differentiation pathway: biochemical-based induction or effects of exposure to mechanical forces of ejection. Deducing the impact of physical cues on cell differentiation fate is difficult to explore within the chemically defined environments of monopotential differentiation media. By using ‘mixed’ media, a bipotential setting was provided whereby the appropriate chemical differentiation cues required for both osteogenesis and adipogenesis were available. This allowed us to study the impact of physical cues, such as mechanical forces or cell carrier, on cell lineage fate independent of chemical dosing variables.

### Impact of cell seeding density on differentiation capacity of hMSCs

No statistically significant differences were detected in hydroxyapatite deposition at the initial seeding densities under investigation. Thus, we postulated that the different initial cell seeding numbers resulting from the various ejection rates tested had negligible effects on mineralisation at day 21, even at the lowest flow rate under investigation (~ 35% of cell dose is delivered at 10 μl/min [28]). These results are consistent with findings from studies carried out on various scaffolds, which demonstrated that higher seeding densities do not necessarily produce enhanced proliferation and differentiation [40, 41]. Holy *et al.* [42] investigated the effect of initial seeding density upon rat bone marrow-derived cell differentiation on PLGA scaffolds and showed that final mineralised tissue formation was independent of initial seeding density.

When cell numbers at day 21 were expressed as fold change in cell number to initial cell seeding number, it was found that lower seeding densities corresponded to the highest proliferation, which agrees with previous studies by McBeath *et al.* [43]. In contrast to the effect on the osteogenesis marker quantified, the adipogenic differentiation capacity was influenced by cell seeding number. This result is in line with previous findings [43, 44]. As mineralisation at day 21 was shown to be independent of initial cell seeding density, osteogenesis was selected as the model for this study. Osteogenesis is a robust, well-established *in-vitro* cellular model of differentiation in hMSCs with well-defined assays and measurable outputs [45].

### Ejection rate influenced osteogenic differentiation capacity of ejected hMSCs

Since we established that the different cell numbers ejected at different rates did not influence total mineralisation levels nor normalised mineralisation-to-cell-number values at day 21, we moved on to investigate the impact of ejection rate on the osteogenic differentiation capacity of ejected hMSCs.

Exposure of MSCs to shear stress, whether controlled (fluid flow) or uncontrolled (flow perfusion), has been reported to result in enhanced osteogenic matrix production and maturation [46–48]. Previous work has qualitatively demonstrated that MSCs ejected at 10 μl/min resulted in considerably lower osteogenic differentiation capacity [28], but no further investigation was carried out. Normalised mineralisation values of cells growing in the bipotential differentiation environment showed a significantly higher level of mineral deposition per cell at the lowest flow rate (10 μl/min) relative to control (Fig. 1b). This suggests that hMSCs ejected at this rate either preferentially underwent osteogenic differentiation relative to the other samples, started differentiating earlier or had enhanced osteogenic efficiency per cell. These differentiation trends confirmed that prolonged exposure to the mechanical forces generated at 10 μl/min, rather than cell density and associated cell–cell interactions, influenced the differentiation fate of ejected cells.

### Biomaterial-assisted delivery

The development and evaluation of convenient, cost-effective and efficient cell delivery systems will aid the translation of cell-based therapies to the clinic. This study explored whether the use of naturally derived biomaterials will influence cellular differentiation and potentially stimulate endogenous regeneration, acting as instructive cell delivery platforms. This study investigated the effects of using various natural biomaterial-based cell carriers for ejection of hMSCs on cell dose recovery, commitment and differentiation capacity in a clinically relevant syringe/needle ejection scenario. The choice of biomaterials reflects our aim to achieve simple, reproducible and clinically relevant delivery of hMSCs to facilitate low-dosage cell therapies.

Since there are unavoidable delays in any clinical cell delivery procedure, cell carriers may provide physical and chemical cues within this time that would inevitably direct cell functionality outcomes, such as proliferation and differentiation. Within biomaterials, cues may include material composition as well as external physical cues resulting from the exposure of cells to mechanical forces associated with injection procedures. Materials utilised in this study were chosen to be readily available, customisable, user-friendly and easily injectable through clinically relevant needles. Since ECM, composed mainly of proteoglycans, glycosaminoglycans and fibrous proteins (e.g. collagen), provides key biochemical and biomechanical cues required for tissue differentiation and homeostasis [49], materials were also selected based on major components of the ECM. Protein-based polymers have the advantage of mimicking characteristics of the natural ECM, and thereby the potential to impact the growth and organisation of transplanted cells [24]. Collagen type I, for example, has been widely applied as scaffolds for cell delivery, including animal models of brain injury, with good biocompatibility [50, 51]. Similarly, gelatin is a hydrolysed form of collagen [52] with biomechanical similarity to the ECM [53]. The supplementation of algal cultures with CMC, as a modifier of interfacial properties, has been reported to protect algal cells against hydrodynamic stress [54].

Results revealed that utilising injectable hydrogels and viscosity modifying excipients for cellular delivery demonstrated positive effects on cell recovery (Fig. 2). A significant loss of cells at 10 μl/min was observed in unprotected (PBS) samples at 24 h post ejection. Gelatin (protein-based) and CMC (polysaccharide-based) cell carriers displayed the highest percentage of viable cells delivered, with no significant difference to directly plated samples. In comparison, collagen and bECM carriers (both protein based) resulted in lower cell recovery, yet were significantly better than ejecting within PBS. An improvement in cell viability post ejection was obtained previously by Aguado *et al.* [10] using alginate gels via a 28G needle, which had been suggested to be due to plug flow, whereby the hydrogel adjacent to the walls undergoes shear thinning and forms a fluid layer which acts as a lubricant [55]. This lubricating fluid layer may be one explanation for the higher percentages of the cell doses delivered in this study through keeping the cells in the central plug zone away from the walls. The significant improvement in cell dose recovery demonstrated herein, in clinically relevant narrow-bore needles at low ejection rates used in clinical trials [35, 56], may be vital to cells that display biological changes after exposure to mechanical forces.

Live/Dead staining confirmed that all tested carriers conserved a high degree of viability of ejected cells. Microscopy revealed a dilute meshwork of what appears to be collagen fibrils dispersed between cells in samples ejected within collagen and bECM, since they were not stained by calcein. Solubilised collagen I can be mixed with living cells during gelation to implant cells in a fibrillar collagen matrix. The force generation and properties of collagen fibrils are comparable to biological filaments, such as actin or microtubules [57]. Non-covalent inter-fibril network interconnections have been reported to transmit cellular traction forces [58]. This may have given rise to extracellular cues that affected cell fate in samples ejected within collagen and bECM relative to hMSCs ejected within PBS. Co-delivery of the cells within the biomaterials studied showed that cells remained viable *in vitro* 10 days post ejection.

Cell dose recovery did not correlate with the biochemical nature of the polymer (protein-based *versus* polysaccharide-based), so rheological studies were carried out to investigate possible correlation of cell recovery with mechanical properties of the biomaterials. Shear-thinning behaviour of these biomaterials, showing a relatively large change in the viscosity (Δ*η* ~ 10^2^–10^4^ Pa.s) from low (0.01 s^− 1^) to high (1000 s^− 1^) shear rates, is a beneficial property for injection-based delivery through narrow-bore needles. Viscometric thixotropy testing revealed that biomaterials were able to recover after shearing, with some biomaterials (collagen and CMC) exhibiting more complete recovery than others. Oscillatory shear rheology of the biomaterials also showed that some of the softer materials with lower storage moduli (G′), such as CMC and gelatin, resulted in better cell recovery than collagen, which has a storage modulus that is 10-fold higher. However, rheological data were not sufficient to explain the significant improvement of cell recovery with gelatin and CMC carriers relative to collagen and bECM. Gelatin and 0.25% CMC, for example, displayed similar storage moduli (Additional file 4: Figure S4), yet showed significantly different cell dose recovery rates. In addition, viscosities and shear-thinning profiles of the two concentrations of CMC under investigation (0.5 and 0.25%) were not significantly different, thereby not explaining the significant difference in dose recovery between them. Therefore, focusing on the rheological properties of a carrier to determine its efficacy at delivering the required number of viable cells is not sufficient.

Surface wettability can influence protein adsorption and, in turn, initial cell attachment. Gelatin resulted in high contact angles on glass despite being structurally similar to collagen due to the preferred orientation of hydrophobic moieties at the air–gel interface, causing a specific arrangement of water molecules [59]. Cell adhesion is similar to physical adhesion in that the cell membrane must make close molecular contact with the surface [60], and therefore we hypothesised that greater levels of cell attachment occurred with surfaces of high wettability since cells can make close contact with these surfaces. Some studies have shown that hydrophilic surfaces produced a significant increase in the amount of protein adsorption, a high initial rate of cell attachment [61, 62] and generally better cell adhesion [63]. It has also been reported that cells adhered and proliferated more on surfaces with moderate hydrophilicity than on the more hydrophobic or hydrophilic spots [64]. Improvement of cell attachment with decreasing contact angles has been observed at incubation times of up to 60 min [62]. The cell carriers with the lowest glass surface wettability in this study displayed the best cell dose recovery. This may be due to the lower contact that these carriers provide with the glass surface of the syringe, discouraging adhesion during the time spent in the delivery device.

Cell recovery trends appeared to correlate with the contact angles displayed by the various carriers, whereby materials displaying the highest contact angles also displayed the highest percentage of cell dose recovered (Figs. 2a and 4b). Moreover, cell recovery did not appear to correlate with the shear-thinning properties of the biomaterials under investigation, as suggested by previous studies [10, 55]. Collagen, for example, showed the best shear-thinning properties yet the lowest percentage of cell dose recovery between the biomaterials tested. The ability of cells to attach to a surface will depend on the cell type and surface used. Further experiments comparing the attachment of different cell types, syringe material surfaces and a wider range of surface wettability values are required to determine the generality of this observation and make use of it to design more efficient cell delivery systems.

A cell’s fate is tightly regulated by its microenvironment, since cells commit to their fate by deriving information from their surroundings [65]. ECM proteins are recognised by cell surface receptors and are involved in cell processes such as proliferation and differentiation. For example, hyaluronan (HA), a naturally occurring polysaccharide found in the ECM of the central nervous system (CNS), can interact with various HA receptors present on diverse cell types to promote cell adhesion and survival [66]. Therefore, it was hypothesised that biomimetic protein-based cell carriers may have a discernible impact on cell commitment and differentiation capacity. Late-stage osteogenic differentiation was enhanced by the protein-based cell carriers, namely collagen, bECM and gelatin, as demonstrated by the mineralisation results. The higher mineralisation levels in samples ejected within collagen (*p* < 0.01*)* compared to control samples at day 21 suggested that the use of a biomimetic cell carrier for the desired cell type can enhance differentiation response significantly. Fibril-forming collagen type I forms more than 90% of the organic mass of bone [67]. Bone marrow MSCs have been reported to undergo osteogenesis when cultured on collagen I matrices *in vitro* by interaction with the COL-I-binding integrin α2β1 [68]. One study hypothesised that adhesion to ECM proteins, in the absence of soluble osteogenic stimulants, can act as insoluble cues of osteogenesis [69]. Given the tissue specificity of ECM and the abundant presence of collagen in bone-derived ECM [70], these protein-based biomaterials have the potential of mimicking native bone microenvironment pre injection and closely mirror the target site once injected [71]. Since a bipotential differentiation environment was used in this study, it could be hypothesised that overall mineralisation was enhanced due to a higher number of cells being directed towards an osteogenic lineage, such as in ECM (high total mineralisation but similar normalised mineralisation levels per cell), and on a ‘mineralisation per cell’ basis in the case of PBS (low total mineralisation but trending towards higher normalised mineralisation levels relative to control). There was no significant difference in hydroxyapatite deposition between directly plated and ejected cells within collagen and bECM at day 21, suggesting that the more extensive mineralisation observed with these carriers relative to PBS is not augmented by mechanical forces encountered by the cells during ejection.

ALP is widely used as an indicator of osteogenic commitment, and commonly precedes bone matrix mineralisation [72, 73]. Significantly higher levels of ALP released into the media were observed in the unprotected samples ejected within PBS relative to directly plated ones. There was also a notable trend of increased media ALP levels in ejected samples relative to directly plated ones within the same bECM carrier. A similar result was previously obtained by Yourek *et al*. [74], whereby 24-h exposure to shear stress resulted in higher ALP activity in the media than in control cells. Results of cellular ALP analysis may be due to mechanical forces caused by the ejection process, resulting in the slower commitment of ejected cells towards the osteogenic lineage *in vitro* (in comparison to direct plating), but stronger osteogenic expression. This is implied by later peaking of normalised ALP expression in ejected samples at day 7, and enhanced mineralisation results with cells ejected within protein-based carriers at day 21. Results suggest that although shear stress in combination with collagen-based carriers supported osteogenic differentiation more effectively relative to the plated control, the commitment process took longer. Correspondingly, Grellier *et al.* [75] exposed hMSCs to short periods of fluid shear stress and showed that 30-min exposure upregulated ALP mRNA but 90-min exposure decreased it to almost basal levels. Moreover, MSCs exposed to oscillatory fluid flow displayed reduced ALP activity despite upregulating OCN mRNA under the same conditions [76]. Osteogenic differentiation is complex and multifactorial, and the detailed mechanism of how ALP acts is unclear [77]. Further studies are needed to explore the impact on differentiation in more detail, since it is reasonable to believe that injectable delivery may impair cells’ ability to differentiate into the required cell type, or cause differentiation into other undesirable cell types.

## Conclusions

This study demonstrated that the use of natural, low-viscosity biomaterials as cell carriers is an efficient approach to significantly improve the percentage of the cell dose delivered relative to the commonly used saline cell vehicle, enabling the administration of low-dosage cell therapies through narrow, clinically relevant needles. This improvement in cell dose recovery was not associated with the rheological properties of the biomaterials utilised, as had been suggested by previous studies.

Moreover, the extent of differentiation in hMSCs, as demonstrated through the use of osteogenesis as a model of stem cell differentiation, was shown to be substantially altered by the selection of biomaterial carrier, despite limited contact time of the carrier with ejected cells during delivery, as well as ejection rate. The development of tailored biomaterial-assisted cell delivery systems for the desired application will accelerate clinical translation of cell-based therapeutics and allow the utilisation of biomaterials for more efficient cell delivery and potentially directing stem cell fate.

## Additional files

Additional file 1: Figure S1. Showing schematic presentation of methodology used to explore effects of various cell carriers on hMSC delivery. Efficacy of delivery, in terms of cell recovery, viability and proliferation capacity, was assessed. In addition, various parameters of osteogenic differentiation were measured to determine the potential impact of various cell carriers on osteogenic differentiation capacity. (PDF 160 kb)

Additional file 2: Figure S2. Showing effect of initial cell seeding density of hMSCs on their osteogenic differentiation potential when cultured in bipotential adipogenic/osteogenic media, quantified based on mineral deposition. (**A**) OsteoImage™ staining for hydroxyapatite in hMSCs from two donors seeded at different initial seeding densities in a 12-well plate, cultured in bipotential media for 21 days (mean ± SD; *n* = 6). No significant difference revealed between various initial cell seeding densities, analysed using one-way ANOVA and Tukey’s *post-hoc* test. ns no significant difference. (**B**) OsteoImage™ fluorescence readings normalised to cell count, based on nuclear staining using PI (mean ± SD, *n*  =  4). Statistical analysis performed using Kruskal–Wallis test with Dunn’s *post-hoc* test. (**C**) PI cell counts normalised to respective initial cell numbers seeded, expressed as fold change relative to initial cell seeding density (mean ± SD, *n* = 4). Data represent averages from two donors. Statistically significant difference from full seeding density of 70,000 cells/well: *p < 0.05, Kruskal–Wallis test with Dunn’s *post-hoc* test. (**D**) Representative fluorescence microscopy images of hMSCs at day 21. Nuclei stained with PI, and hydroxyapatite stained fluorescently using OsteoImage™ (scale bar = 100 μm). (PDF 1007 kb)

Additional file 3: Figure S3. Showing effect of initial cell seeding density of hMSCs on their adipogenic differentiation when cultured in bipotential adipogenic/osteogenic media. (**A**) AdipoRed™ staining for lipid content in hMSCs seeded at different initial seeding densities in a 12-well plate, cultured in bipotential media for 21 days (*n* = 4). Statistically significant difference from the full seeding density of 70,000 cells/well: **p < 0.01, *p < 0.05, Kruskal–Wallis test with Dunn’s post-hoc test. (**B**) AdipoRed™ fluorescence readings, adjusted for cellular count based on nuclear staining using Hoechst 33,258 (mean ± SD, *n*  =  3 in triplicates). Statistical analysis performed using Kruskal–Wallis test with Dunn’s *post-hoc* test. *p < 0.05. (**C**) Fluorescence microscopy images of hMSCs cultured in bipotential differentiation media at day 21. Lipid droplets stained fluorescently using AdipoRed™ Adipogenesis Reagent, after which nuclei were counterstained with Hoechst (scale bar = 100 μm). (PDF 610 kb)

Additional file 4: Figure S4. Showing oscillatory rheological measurements of biomaterial-based carriers to obtain storage (G′) and loss (G″) moduli from a strain-amplitude sweep (0.1–1000%) performed at 6 rad/s (*n* ≥ 3). Carried out for (**A**) 5 (0.5%) and 2.5 (0.25%) mg/ml CMC, (**B**) 20 mg/ml (2%) gelatin, (**C**) 1.75 mg/ml collagen and (**D**) 1.75 mg/ml bone ECM. (PDF 153 kb)

Additional file 5: Figure S5. Showing cellular ALP activity levels of hMSCs at different time points following ejection at 10 μl/min via 30G needles, and cultured in bipotential media. (**A**) Cellular ALP analysed 2, 4 and 7 days post induction. Values are mean ± SD (*n* = 3 in two donors). Statistically significant differences in ALP levels relative to control (Friedman test with Dunn’s post-hoc test: *p < 0.05. (**B**) Cellular ALP values normalised to DNA content (mean ± SD, *n* = 3 in two donors). (**C**) Normalised cellular ALP levels in ejected versus directly plated hMSCs suspended within collagen and ECM. ‘Ejected’ cells ejected at 10 μl/min, and ‘plated’ cells were 60% of the initial cell number directly plated (mean ± SD, *n*  =  3 in two donors). (**D**) DNA content of hMSCs in ejected versus directly plated samples suspended within collagen and ECM (mean ± SD). (**E**) Representative immunofluorescent staining of human osteocalcin (OCN) and nuclei counterstained with DAPI (blue) to confirm osteogenic differentiation of hMSCs. Directly plated and ejected hMSCs (via 30G needles at 10 μl/min) cultured in bipotential media at 21 days post induction (scale bar = 50 μm). (PDF 926 kb)

## Abbreviations

ALP: Alkaline phosphatase
bECM: Bone extracellular matrix
hMSC: Human mesenchymal stem cell
OCN: Osteocalcin
PBS: Phosphate buffered saline
