# Supplementary material for: A biomaterials approach to influence stem cell fate in injectable cell-based therapies
Source: Stem Cell Res Ther. 2018 Feb 21;9:39. doi: 10.1186/s13287-018-0789-1 (PMC5822649; doi:10.1186/s13287-018-0789-1)
Supplement: Supplementary file 1 — Showing schematic presentation of methodology used to explore effects of various cell carriers on hMSC delivery. Efficacy of delivery, in terms of cell recovery, viability and proliferation capacity, was assessed. In addition, various parameters of osteogenic differentiation were measured to determine the potential impact of various cell carriers on osteogenic differentiation capacity. (PDF 160 kb) [file 13287_2018_789_MOESM1_ESM.pdf]

Additional file 1: Figure S1

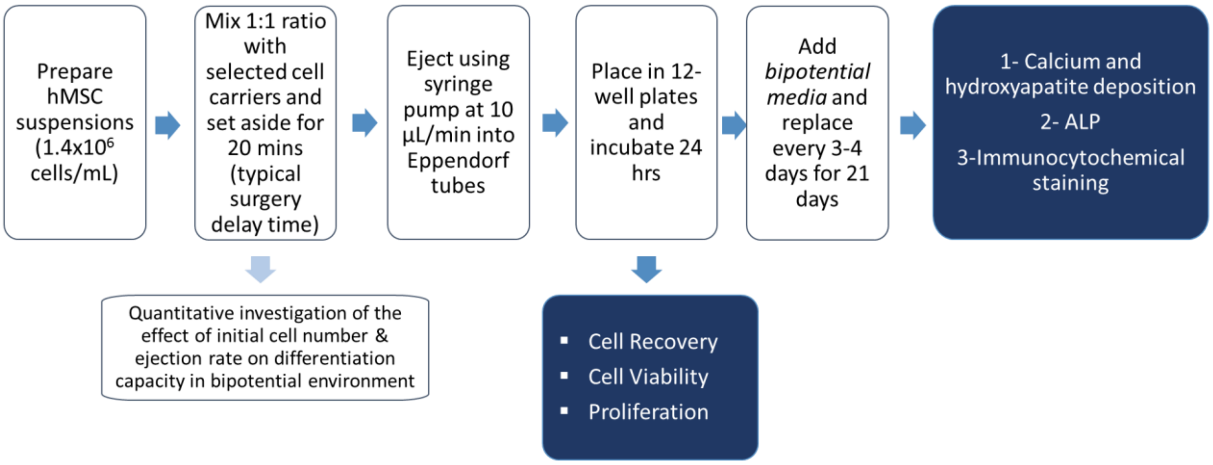

Fig. S1: **Schematic presentation of the methodology used to explore the effects of various cell carriers on hMSC delivery.** Efficacy of delivery, in terms of cell recovery, viability and proliferation capacity, was assessed. In addition, various parameters of osteogenic differentiation were measured to determine the potential impact of various cell carriers on osteogenic differentiation capacity.
