# Supplementary material for: A biomaterials approach to influence stem cell fate in injectable cell-based therapies
Source: Stem Cell Res Ther. 2018 Feb 21;9:39. doi: 10.1186/s13287-018-0789-1 (PMC5822649; doi:10.1186/s13287-018-0789-1)
Supplement: Supplementary file 2 — Showing effect of initial cell seeding density of hMSCs on their osteogenic differentiation potential when cultured in bipotential adipogenic/osteogenic media, quantified based on mineral deposition. (A) OsteoImage™ staining for hydroxyapatite in hMSCs from two donors seeded at different initial seeding densities in a 12-well plate, cultured in bipotential media for 21 days (mean ± SD; n = 6). No significant difference revealed between various initial cell seeding densities, analysed using one-way ANOVA and Tukey’s post-hoc test. ns no significant difference. (B) OsteoImage™ fluorescence readings normalised to cell count, based on nuclear staining using PI (mean ± SD, n = 4). Statistical analysis performed using Kruskal–Wallis test with Dunn’s post-hoc test. (C) PI cell counts normalised to respective initial cell numbers seeded, expressed as fold change relative to initial cell seeding density (mean ± SD, n = 4). Data represent averages from two donors. Statistically significant difference from full seeding density of 70,000 cells/well: *p < 0.05, Kruskal–Wallis test with Dunn’s post-hoc test. (D) Representative fluorescence microscopy images of hMSCs at day 21. Nuclei stained with PI, and hydroxyapatite stained fluorescently using OsteoImage™ (scale bar = 100 μm). (PDF 1007 kb) [file 13287_2018_789_MOESM2_ESM.pdf]

Additional file 2: Figure S2

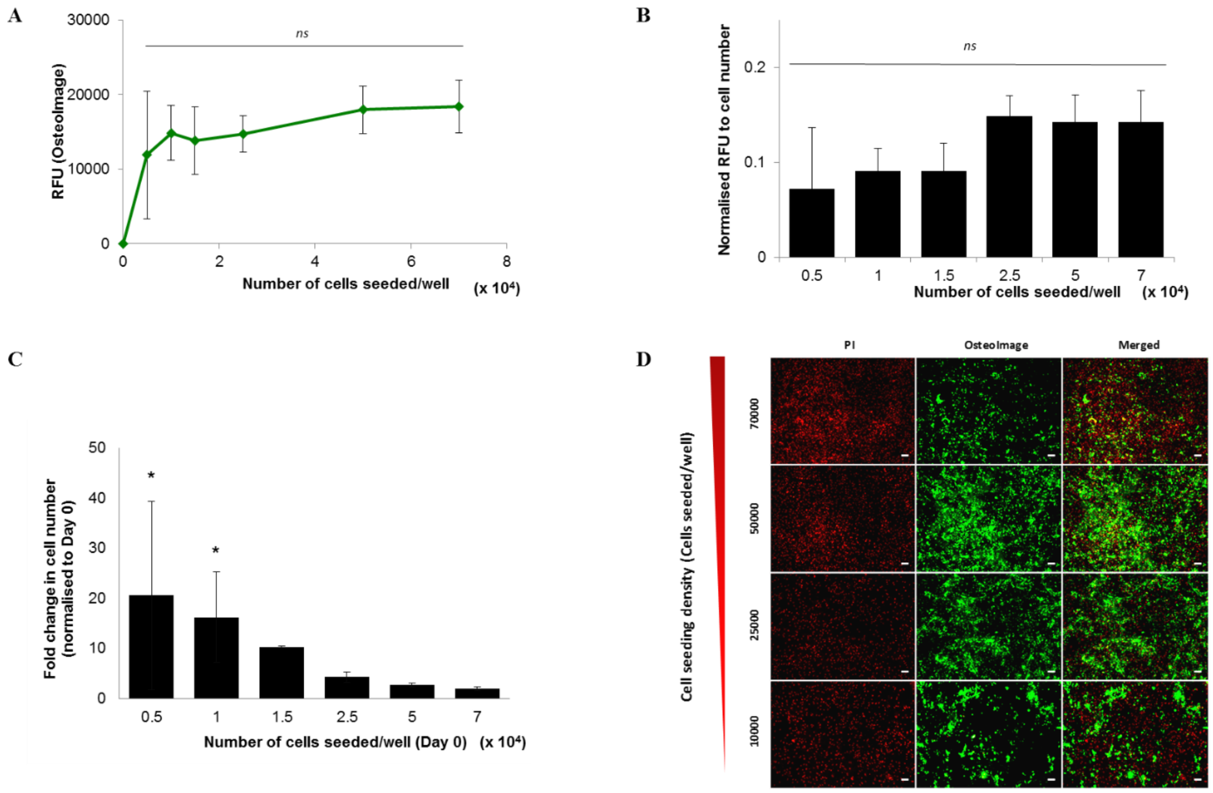

Figure S2: **Effect of initial cell seeding density of hMSCs on their osteogenic differentiation potential when cultured in bipotential adipogenic/osteogenic media, quantified based on mineral deposition.** (A) OsteoImage staining for hydroxyapatite in hMSCs from two donors seeded at different initial seeding densities in a 12-well plate, cultured in bipotential media for 21 days (mean ± SD, *n*=6). No significant difference was revealed between the various initial cell seeding densities, analysed using one-way ANOVA and Tukey's *post-hoc* test (B) OsteoImage fluorescence readings were normalised to cell count, based on nuclear staining using PI (mean ± SD, *n*=4). Statistical analysis was performed using Kruskal-Wallis test, with Dunn's *post-hoc* test; *ns*: no significant difference. (C) PI cell counts normalised to the respective initial cell numbers seeded, expressed as the fold change relative to initial cell seeding density (mean ± SD, *n*=4). Data represents averages from two donors; Asterisks indicate statistically significant difference from full seeding density of 70,000 cells/well (**p*<0.05 - Kruskal-Wallis test, with Dunn's *post-hoc* test). (D) Representative fluorescence microscopy images of hMSCs at day 21. Nuclei were stained with PI, and hydroxyapatite was fluorescently stained using OsteoImage (Scale bar=100 µm).
