# Supplementary material for: A biomaterials approach to influence stem cell fate in injectable cell-based therapies
Source: Stem Cell Res Ther. 2018 Feb 21;9:39. doi: 10.1186/s13287-018-0789-1 (PMC5822649; doi:10.1186/s13287-018-0789-1)
Supplement: Supplementary file 3 — Showing effect of initial cell seeding density of hMSCs on their adipogenic differentiation when cultured in bipotential adipogenic/osteogenic media. (A) AdipoRed™ staining for lipid content in hMSCs seeded at different initial seeding densities in a 12-well plate, cultured in bipotential media for 21 days (n = 4). Statistically significant difference from the full seeding density of 70,000 cells/well: **p < 0.01, *p < 0.05, Kruskal–Wallis test with Dunn’s post-hoc test. (B) AdipoRed™ fluorescence readings, adjusted for cellular count based on nuclear staining using Hoechst 33,258 (mean ± SD, n = 3 in triplicates). Statistical analysis performed using Kruskal–Wallis test with Dunn’s post-hoc test. *p < 0.05. (C) Fluorescence microscopy images of hMSCs cultured in bipotential differentiation media at day 21. Lipid droplets stained fluorescently using AdipoRed™ Adipogenesis Reagent, after which nuclei were counterstained with Hoechst (scale bar = 100 μm). (PDF 610 kb) [file 13287_2018_789_MOESM3_ESM.pdf]

# Additional file 3: Figure S3

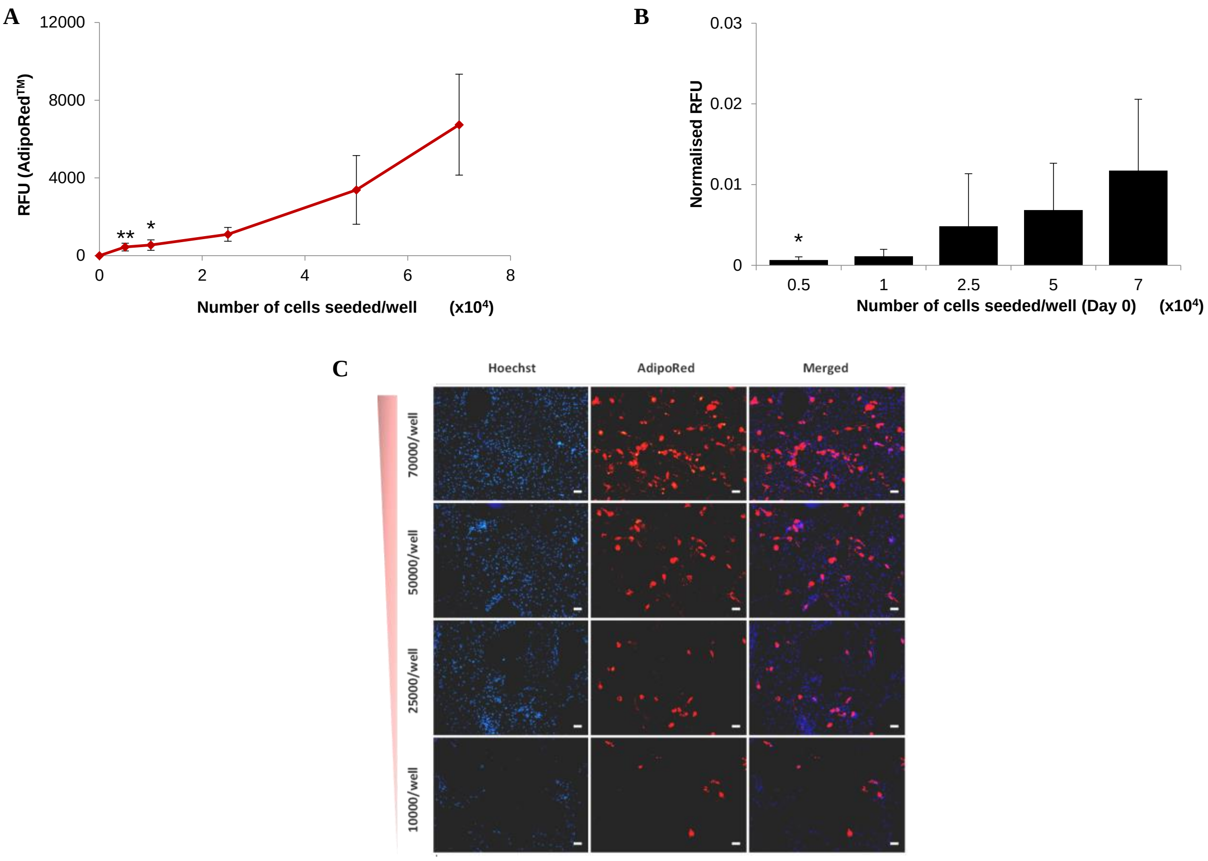


Figure S3: **Effect of initial cell seeding density of hMSCs on their adipogenic differentiation when cultured in bipotential adipogenic/osteogenic media.** (A) AdipoRed staining for lipid content in hMSCs seeded at different initial seeding densities in a 12-well plate, cultured in bipotential media for 21 days ($n$=4). Asterisks indicate statistically significant difference from the full seeding density of 70,000 cells/well (**$p$<0.01, *$p$<0.05 - Kruskal-Wallis test, with Dunn's *post-hoc* test). (B) AdipoRed fluorescence readings, adjusted for cellular count based on nuclear staining using Hoechst 33258 (mean ± SD, $n$=3 in triplicates). Statistical analysis was performed using Kruskal-Wallis test, with Dunn's *post-hoc* test (*$p$<0.05). (C) Fluorescence microscopy images of hMSCs cultured in bipotential differentiation media at day 21. Lipid droplets were fluorescently stained using AdipoRed Adipogenesis Reagent, after which nuclei were counter-stained with Hoechst. (Scale bar=100 µm).
