# Supplementary material for: A biomaterials approach to influence stem cell fate in injectable cell-based therapies
Source: Stem Cell Res Ther. 2018 Feb 21;9:39. doi: 10.1186/s13287-018-0789-1 (PMC5822649; doi:10.1186/s13287-018-0789-1)
Supplement: Supplementary file 4 — Showing oscillatory rheological measurements of biomaterial-based carriers to obtain storage (G′) and loss (G″) moduli from a strain-amplitude sweep (0.1–1000%) performed at 6 rad/s (n ≥ 3). Carried out for (A) 5 (0.5%) and 2.5 (0.25%) mg/ml CMC, (B) 20 mg/ml (2%) gelatin, (C) 1.75 mg/ml collagen and (D) 1.75 mg/ml bone ECM. (PDF 153 kb) [file 13287_2018_789_MOESM4_ESM.pdf]

Additional file 4: Figure S4

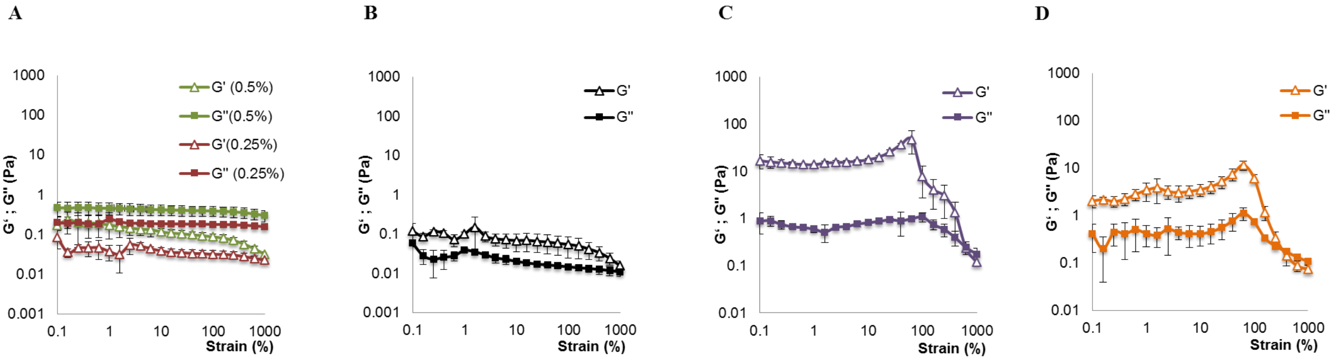

Figure S4: **Oscillatory rheological measurements of the biomaterial-based carriers to obtain storage (G') and loss (G'') moduli from a strain-amplitude sweep (0.1–1000%) performed at 6 rad/s (*n*≥3).** This was carried out for (A) 5 (0.5%) and 2.5 (0.25%) mg/mL CMC, (B) 20 mg/mL (2%) gelatin, (C) 1.75 mg/mL collagen, (D) 1.75 mg/mL bone ECM.
