# Supplementary material for: A biomaterials approach to influence stem cell fate in injectable cell-based therapies
Source: Stem Cell Res Ther. 2018 Feb 21;9:39. doi: 10.1186/s13287-018-0789-1 (PMC5822649; doi:10.1186/s13287-018-0789-1)
Supplement: Supplementary file 5 — Showing cellular ALP activity levels of hMSCs at different time points following ejection at 10 μl/min via 30G needles, and cultured in bipotential media. (A) Cellular ALP analysed 2, 4 and 7 days post induction. Values are mean ± SD (n = 3 in two donors). Statistically significant differences in ALP levels relative to control (Friedman test with Dunn’s post-hoc test: *p < 0.05. (B) Cellular ALP values normalised to DNA content (mean ± SD, n = 3 in two donors). (C) Normalised cellular ALP levels in ejected versus directly plated hMSCs suspended within collagen and ECM. ‘Ejected’ cells ejected at 10 μl/min, and ‘plated’ cells were 60% of the initial cell number directly plated (mean ± SD, n = 3 in two donors). (D) DNA content of hMSCs in ejected versus directly plated samples suspended within collagen and ECM (mean ± SD). (E) Representative immunofluorescent staining of human osteocalcin (OCN) and nuclei counterstained with DAPI (blue) to confirm osteogenic differentiation of hMSCs. Directly plated and ejected hMSCs (via 30G needles at 10 μl/min) cultured in bipotential media at 21 days post induction (scale bar = 50 μm). (PDF 926 kb) [file 13287_2018_789_MOESM5_ESM.pdf]

Additional file 5: Figure S5

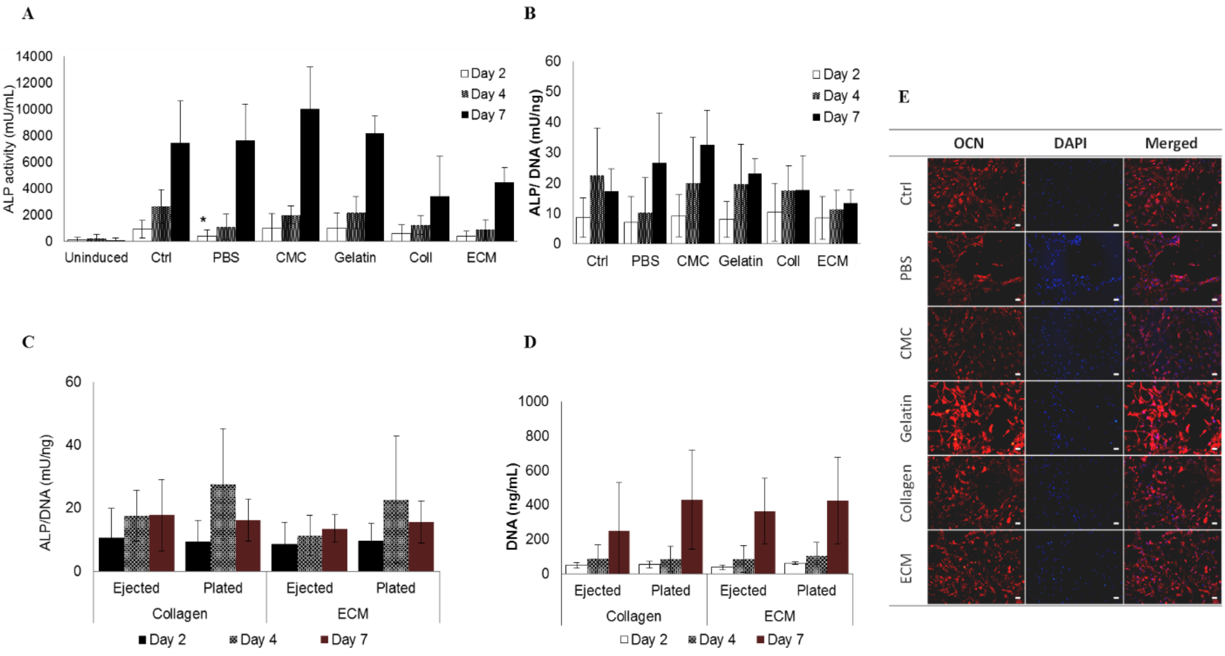

Figure S5: **Cellular alkaline phosphatase activity levels of hMSCs at different time points following ejection at 10 µL/min via 30G needles, and cultured in bipotential media.** (A) Cellular ALP was analysed 2, 4 and 7 days post-induction. Values shown are mean ± SD (*n*=3; 2 donors). Asterisks indicate statistically significant differences in ALP levels relative to control (Friedman test with Dunn's *post-hoc*; *$p<0.05$) (B) Cellular ALP values normalised to DNA content (mean ± SD; *n*=3; 2 donors) (C) Normalised cellular ALP levels in ejected versus directly-plated hMSCs suspended within collagen and ECM. "Ejected" cells were ejected at 10 µL/min, and "plated" cells were 60% of the initial cell number directly plated (mean ± SD; *n*=3 in 2 donors). (D) DNA content of hMSCs in ejected versus directly plated samples suspended within collagen and ECM (mean ± SD). (E) Representative immunofluorescent staining of human osteocalcin (OCN) and nuclei counterstained with DAPI (blue) to confirm osteogenic differentiation of hMSCs. Directly plated and ejected hMSCs (via 30G needles at 10 µL/min), were cultured in bipotential media at 21 days post-induction (Scale bar = 50 µm).
